# How to sleep as a woman with ADHD and autism spectrum disorder

**DOI:** 10.1192/j.eurpsy.2026.10311

**Published:** 2026-07-31

**Authors:** D. Wynchank

**Affiliations:** 1 ADHD in Adults, PsyQ, The Hague; 2 Psychiatry, Amsterdam UMC, Amsterdam, Netherlands

## Abstract

**Introduction:**

Sleep problems are highly prevalent in adults with neurodevelopmental disorders, with evidence of sex- and age-specific patterns. In women with ADHD, hormonal fluctuations across the reproductive lifespan interact with executive dysfunction, increasing vulnerability to sleep and cognitive disturbances. Women with ADHD report more severe sleep problems than men, including higher rates of insomnia and hypersomnia. Sleep disruption is also highly prevalent in autism spectrum disorder (ASD), affecting up to 80% of women, who are particularly prone to insomnia, circadian rhythm disturbances, and heightened sleep reactivity. Recent findings suggest poorer subjective sleep quality and more fragmented sleep, in autistic women, although age-related data remain limited. Menopausal transition may further compound these difficulties.

**Objectives:**

To illustrate how perimenopausal hormonal transitions exacerbate sleep difficulties in women with ADHD and ASD through a case-based approach. To integrate clinical and scientific evidence highlighting the need for age- and sex-sensitive strategies in the management of neurodevelopmental disorders across the female lifespan.

**Methods:**

This clinical vignette describes a 46-year-old woman with combined-type ADHD and ASD, who was referred for evaluation of persistent sleep difficulties during the perimenopausal transition. Diagnostic assessment included the DIVA-5, ADOS-2, RAADS-R, developmental history, collateral informant report, structured questionnaires (HSDQ, mood and anxiety), actigraphy, and clinical evaluation of hormonal status.

**Results:**

The patient had longstanding ADHD and autistic traits. Perimenopause was marked by worsening insomnia, delayed sleep phase, and nocturnal awakenings with hot flushes. Actigraphy confirmed circadian disruption and frequent awakenings. Cognitive testing showed decline in working memory and planning. Sleep disturbance amplified anxiety, irritability, and neurodevelopmental symptoms. Methylphenidate improved attention but worsened sleep; lisdexamfetamine was better tolerated. Melatonin improved sleep timing but not maintenance. Menopausal hormone therapy (transdermal oestradiol, oral micronised progesterone) improved vasomotor symptoms but not sleep fragmentation.

**Conclusions:**

This case illustrates how perimenopause can exacerbate sleep problems in women with ADHD and ASD. Literature supports high rates of insomnia and circadian rhythm disruption in both groups, with female-specific vulnerabilities. Recognition of reproductive transitions as periods of heightened risk is essential. Age- and sex-sensitive strategies may improve cognition, emotional wellbeing, and quality of life in women with neurodevelopmental disorders.

**Disclosure of Interest:**

None Declared

